# 

**Published:** 1871-01

**Authors:** 


### INDEX TO VOLUME XVIII.

| | PAGE |
|---|---|
| Asthma | 37 |
| Author's Experience | 262 |
| A Golden Child | 278 |
| Biliousness | 44 |
| Benevolent Men | 110 |
| Born Perfect | 113 |
| Bad Colds | 121 |
| Breath Measured | 129 |
| Barbers | 133 |
| Bad Smell | 164 |
| Bath Towel | 227 |
| Blood Tells | 233 |
| Carbolic Acid | 12 |
| Cities, Population of | 21 |
| Coal Fires | 25 |
| Chill and Fever | 27, 251 |
| Coffee | 33, 116 |
| Complaining | 33 |
| Consumptives' Home | 62, 95, 202 |
| Corns | 92 |
| Country People | 102 |
| Curiosities of Life | 114 |
| Colds | 121 |
| Candies | 132 |
| Cholera | 138, 245 |
| Catching Cold | 184, 198 |
| Calico | 184 |
| Cooking Economies | 188 |
| Carpets | 189 |
| Clergymen and Jay Cooke | 197 |
| Children, Church | 204 |
| Cancer | 212 |
| Chloral Hydrate | 255 |
| Competition | 259 |
| Character | 265 |
| Cure for a Felon | 277 |
| Domestic Secret | 8 |
| Desserts | 10 |
| Disinfectants | 12, 172 |
| Dyspeptic | 15, 149, 238 |
| Drunkenness | 53, 85 |
| Dancing | 59 |
| Doctor, Best | 75 |
| Death, Causes of | 143 |
| Death Damps | 191 |
| Dumb | 201 |
| Diabetes | 232 |
| Envelopes, Mourning | 104 |
| Eggs, Keeping | 111, 164 |
| Eating | 131, 154, 157 |
| Editors, Brave | 166 |
| Ear Diseases | 207 |
| Educated for Nothing | 271 |
| Emergencies | 274 |
| Feet | 7 |
| Fever and Ague | 39 |
| Family Reading | 63 |
| Fun | 65, 134 |
| Failure, Business | 81 |
| Floors, Cold | 113 |
| Finger-nails, Black | 125 |
| Fence the Family | 145 |
| Foundlings | 159 |
| Fruits, Canned | 228 |
| Flowers and Health | 243 |
| Fruits and Berries | 269 |
| Going Abroad | 90, 131 |
| Getting Wet | 156 |
| Girl Training | 180 |
| Green Corn | 225 |
| Games | 259 |
| Gases | 260 |
| Hiccough | 40 |
| House Warming | 51 |
| Hair Dyes | 56 |
| Hall, some other | 57 |
| Houses, Cheap | 153 |
| Honey, Preserved | 175 |
| Hunger | 186 |
| Holiness is Health | 255 |
| Hog Cholera | 261 |
| Human Scalp | 278 |
| Itch | 80 |
| Independent Men | 83 |
| Innocents, Murdered | 88 |
| Ignorance and Disease | 141 |
| Ice Cream | 183 |
| Insanity | 223 |
| Ice-Houses | 273 |
| Indefinites | 260 |
| Jerusalem | 229 |
| Kerosene Dangers | 89, 216 |

PAGE

Lucky People.................... 13
Learned Doctors.................. 19
Lungs, Inflammation of....... 20, 244
Liver........................ 43, 199
Longest Livers.............. 115, 175
Lead Water-Pipes...... 127, 184, 241
Left-Handedness.................. 186
Life's Chances................... 198
Life-Preservers.................. 204
Liquor Drinking.................. 216
Lightning Rods................... 233
Mental and Moral Might........... 1
Mental Recreation................ 279
Mechanics........................ 57
Milk, Unwholesome................ 161
Mind Kills....................... 127
Miasm............................ 138
Medicines, Best.................. 160
Names, Honored................... 28
Nose Drenching................... 47
No and Yes....................... 59
Newspaper Influences....... 107, 146
Nursing.......................... 136
Neighborly....................... 257
Nervousness...................... 258
Overworked................. 162, 193
Old Men, Poor.................... 210
Old Maids........................ 215
Pies and Pastries................ 10
Purifiers........................ 12
Physicians, Educated............. 20
Pneumonia........................ 20
"Pathies," All................... 55
Patent Medicines................. 86
Prisoners' Health................ 155
Preaching.................. 178, 196
Poor, The Dying.................. 204
Paintings Cleaned................ 222
Pin-worms........................ 261
Roll of Honor.................... 28
Rice............................. 30
Rich People...................... 97
Reading, Dangerous............... 145
Ruts of Life..................... 161
Rest............................. 226
Religious, A Daily............... 235
Suicide..................... 31, 157
Suppers, Hearty.................. 41
Sick and Poor.................... 45
Sleep................. 49, 133, 254
Singing, Church.................. 76

PAGE

Servants......................... 80
Spring Dangers................... 88
Summer Excursions........... 91, 93
Starving......................... 94
Summer Drinks......... 105, 128, 213
Small-Pox........................ 108
Sorrow Kills..................... 112
Shaving.......................... 133
Sea-Bathing...................... 152
Sun-stroke................. 167, 177
Sunday Dinner.................... 179
Soda Water....................... 181
Success.......................... 181
Shocks........................... 187
Saving Strength.................. 208
School-girls............... 213, 225
School System.................... 218
Sleeping Together................ 247
Softening of the Brain........... 272
Tyng, S. H....................... 4
Thermometers..................... 18
Tooth-picks...................... 35
Tomatoes.................... 39, 227
Tea and Coffee................... 116
Travel...................... 90, 131
Trichinæ......................... 137
Travelling Thoughts.............. 232
Temperance....................... 249
Tape-Worms....................... 261
Uniformities, Nature's........... 187
Voice............................ 5
Velocities....................... 84
Vaccination................ 136, 209
Ventilation................ 173, 253
Vegetable Remedies............... 211
Winter Shoes..................... 37
Water-Closets.................... 42
Warming Houses................... 51
Writing Restored................. 83
Workmen.......................... 109
Write Plainly.................... 113
Weight, Height, Breath........... 129
Whiskey Work..................... 132
Worked to Death............ 162, 193
Water-Pumps...................... 170
Water-Pipes...................... 184
Whitewashing..................... 228
"Witness, New York".............. 235
Wood Fires Again................. 281
Yes and No....................... 59
Young Men, Rules for............. 234

### BOOK NOTICES.

"The Home Journal" is the handsomest weekly newspaper in the United States, in the world. On entering one of our city mansions for the first time, a stranger to its occupants, we feel at home the moment we see the "Home Journal" on the table, for its very presence is a demonstration that there is grace, and elegance, and culture, and refinement in that household; that it is the home of art and music, and a high civilization.

As evidence of its public appreciation, the publishers commence the new year with eight more columns of reading, without any increase in price; this is a great advance in its prosperous career, which will doubtless be met with a corresponding increase of public patronage.

LEE & SHEPARD's Publications, New York and Boston. These indefatigable publishers, the special friends of children, have issued "Little Prudy's Fly-away Series," No. 1; "Little Folks Astray," by SOPHIE MAY, illustrated. Also, "Light at Eventide," in beautiful style, being a compilation of choice Religious Hymns and Poems, by the editor of "Chimes for Childhood," — DANE ESTES. Among the selections of this volume we observe some of the most beautiful hymns in the languages, with the authors' names and index of first lines. Also, "Springdale Stories," six vols., in beautiful case: "Adele;" "Nettie's Trial;" "Herbert;" "Eric Johnstone's Farm;" "Ennisfallen," beautifully illustrated. Such of our readers as want to gladden the eyes and hearts of their children for the holidays should by all means call on Lee & Shepard: Boston and New York.

"Piano and Musical Matter," by G. DE LA MOTTE, now in its fourth edition, evidencing public appreciation, by LEE & SHEPARD, Boston, Mass. Also, by same publishers, "Letters Everywhere," being stories and rhymes for children. Also, "Why and How," by RUSSEL H. CONWELL, showing why the Chinese emigrate, and the means they adopt for the purpose of reaching America, with sketches of travel, amusing incidents, social customs, etc. Also, "The Social Stage; or, Original Dramas, Comedies, Burlesques, Entertainments," etc., a book which we do not advise any one to purchase, because it is not worth shelf room.

LEE & SHEPARD's Publications, Boston and New York. "The Beckoning Stories," illustrated: "Who will Win," by PAUL COBDEN, illustrating the text, "All must run the race of life, and as on no other race-course, all who will, may win;" "Going on a Mission," by same author, and beautifully illustrated; text, "Without crossing deep seas or broad oceans, all who will may go on a mission" for good.

Then comes a beautiful volume of the "Onward and Upward Mission," by OLIVER OPTIC, with fourteen illustrations.

ZELL's POPULAR ENCYCLOPEDIA, being a Universal Dictionary of English Language, Science, Literature and Art, by L. COLANGE, LL. D. Two vols., illustrated by over 2,000 wood-cuts, by T. ELLWOOD ZELL, Philadelphia, Penn., and agency, 5 Beekman St., New York, Mr. B. W. Bond, manager. No encyclopedia yet published has been received with more favor by the public press throughout the whole country. Its first quality is its reliability; second, succinctness in treatment; and third, the wide range of subjects. It is published in monthly numbers, at fifty cents each, so that it can be paid for easily by almost all persons of education and culture. The first volume is completed and substantially bound, of 1,196 pages quarto, three closely printed columns on each page; hence for the price, it contains a vast amount of information from A to H. The other numbers remaining will appear with great promptitude.

"The People's Literary Companion," 75 cents a year, by E. C. ALLEN & Co., Augusta, Maine. A very beautiful engraving is sent free to every subscriber; the one entitled "From Shore to Shore," is a delightful picture for study; its suggestiveness makes it worth more than the subscription price.

"The Pitcher of Cool Water" and other stories, by T. S. ARTHUR, is another of the excellent publications of THE NATIONAL TEMPERANCE SOCIETY, 17[illegible] William St., New York.

"The House on Wheels, or the Stolen Child," translated from the French by Miss E. F. Adams, with twenty illustrations by Emile Bayard. The scenes are laid in Normandy, and are well described, with many striking incidents.

"Health by Good Living." Henry T. Tuckerman writes as follows to the *Boston Transcript*: —

"In New York the practical precepts in regard to 'Health by Good Living,' contained in Dr. Hall's book with that title, published by Hurd & Houghton, have produced a remarkable change in the habits of many people, who declared that they have got rid of their ailments by following this writer's judicious advice. We have heard of a lady eighty years old, who declares that she has not been so well for twenty years; of a gouty *bon vivant* who has resorted to regular and moderate habits, and is thereby rejuvenated; and of a pale and fair devotee of fashion who has left off eating *confetti*, and recovered her bloom. No wonder the publishers sold fifteen thousand copies of the book in a few weeks."

"Coughs and Colds; or, The prevention, cause, and cure of various affections of the throat, with cases illustrating the remarkable efficacy of outdoor activity and horseback exercise in permanently arresting the progress of diseases of the chest." This volume is published by Hurd & Houghton, 13 Astor Place, New York, and with "Health by Good Living," may be found at the principal bookstores of the country. Price $1.50.

The New York "Independent" of December 1st, says: — "Dr. Hall's 'Coughs and Colds' has a title-page so long that we cannot copy it; but, if we made this notice ten times as long as Dr. Hall's title, we could not begin to express our appreciation of this sensible, admirable, wise book about lung diseases."

Useful Prose. — Dr. Hall's "Coughs and Colds" is a thoroughly practical volume, giving a common-sense view of the commonest (and in some respects most dangerous) disease in the world, and the methods of its prevention and cure. It ought to save many lives, and it will, if those immediately concerned will let it do so. — *Congregationalist*, November, 1870.

The demand for "Rand's Sea Moss Farine" in this country and Great Britain is greater than the present manufactory can promptly supply.

### Horace Greeley once said, with great truth, that to have notable success in New York, one man must have the power to do the work of three. This is creditably demonstrated in the career of a merchant who, three years ago, thought he was doing well if he sold a few hundred dollars worth of goods in a week. On the 19th of November he sold at retail over eleven thousand dollars worth, having often before sold ten thousand worth a day. Baldwin, the Clothier, is half a dozen men in one, — a tailor in business, a courtier in manners, a gentleman by nature; in addition he is the editor of his own paper, a writer, and a very Napoleon in the marshalling of his business forces and the army of men under him.

Dr. Hall's office hours, for medical consultation only, are from 10 to 2 daily, at No. 2 West 43d Street, New York, corner of Fifth Avenue. Will be found at 176 Broadway, Room 8, from 3 to 4 on Tuesdays, Thursdays, and Saturdays Persons coming for advice or business will not be admitted to his private residence.

